# Protein intake in early childhood and cardiometabolic health at school age: the Generation R Study

**DOI:** 10.1007/s00394-015-1026-7

**Published:** 2015-09-02

**Authors:** Trudy Voortman, Edith H. van den Hooven, Myrte J. Tielemans, Albert Hofman, Jessica C. Kiefte-de Jong, Vincent W. V. Jaddoe, Oscar H. Franco

**Affiliations:** 1 Department of Epidemiology, Erasmus MC, University Medical Center, Rotterdam, The Netherlands; 2 The Generation R Study Group, Erasmus MC, University Medical Center, Rotterdam, The Netherlands; 3 Department of Pediatrics, Erasmus MC, University Medical Center, Rotterdam, The Netherlands

**Keywords:** Dietary protein, Children, Body fat, Blood pressure, Insulin, Epidemiology

## Abstract

**Purpose:**

High protein intake in infancy has been linked to obesity. We aimed to examine the associations of protein intake in early childhood with cardiovascular and metabolic outcomes at school age.

**Methods:**

This study was performed in 2965 children participating in a population-based prospective cohort study. Protein intake at 1 year was assessed with a food frequency questionnaire and was adjusted for energy intake. At the children’s age of 6 years, we measured their body fat percentage (BF%), blood pressure (BP), and insulin, HDL cholesterol, and triglyceride serum levels. These measures were incorporated into a cardiometabolic risk factor score, using age- and sex-specific SD scores.

**Results:**

In covariate-adjusted models, higher protein intake was associated with a higher BF%, lower diastolic BP, and lower triglyceride levels. We observed a significant interaction of protein intake with child sex on metabolic outcomes. Stratified analyses showed that protein intake was positively associated with BF% [0.07 SD (95 % CI 0.02; 0.13) per 10 g/day] and insulin levels in girls, but not in boys. In boys, but not in girls, higher protein intake was associated with lower triglyceride levels [−0.12 SD (95 % CI −0.20; −0.04) per 10 g/day] and a lower cardiometabolic risk factor score. Protein intake was not consistently associated with systolic BP or HDL cholesterol levels.

**Conclusion:**

Protein intake in early childhood was associated with a higher BF% and higher insulin levels at 6 years in girls and with lower triglyceride levels in boys. Further studies are needed to explore these sex differences and to investigate whether the observed changes persist into adulthood.

**Electronic supplementary material:**

The online version of this article (doi:10.1007/s00394-015-1026-7) contains supplementary material, which is available to authorized users.

## Introduction

Already in childhood, adiposity, high blood pressure, dyslipidemia, and insulin resistance are highly prevalent [1]. These cardiometabolic risk factors in childhood have been shown to track to later life and are suggested to predict adult cardiovascular disease and type 2 diabetes [2, 3], highlighting the need to study determinants of cardiometabolic health already in early childhood [4].

Studies in adults suggest beneficial effects of high dietary protein intake on cardiometabolic risk factors, including a lower blood pressure, lower triglyceride levels, and a reduction in body weight [5–7]. Mechanisms underlying these effects remain to be elucidated, but may include increased satiety, increased energy expenditure, and metabolic effects of specific amino acids [8]. In contrast, a high protein intake in early childhood has been associated with a higher risk of obesity [9–12], suggesting that high protein intake in early life may lead to unfavorable effects on cardiometabolic health. A high protein intake in infancy may enhance the secretion of insulin-like growth factor 1 (IGF-1) and insulin [13, 14], which could in turn increase adipogenesis [15]. The period around the age of 1 year has been suggested to be a critical phase with respect to protein intake and later obesity risk, possibly because this period is often characterized by a transition from complementary feeding to family diet and a corresponding rapid increase in protein intake [16]. Thus far, studies investigating the effects of protein intake on insulin levels, blood lipids, and blood pressure in children are scarce and report inconsistent results [17].

We examined the associations of protein intake at the age of 1 year with body fat percentage, insulin levels, blood lipids, and a combined cardiometabolic risk factor score at the age of 6 years in 2965 children participating in a population-based prospective cohort study. In addition, we aimed to evaluate whether the associations differed for different sources of protein and whether associations differed by child sex, ethnicity, birth weight, or weight status at 6 years.

## Subjects and methods

### Study population

**Fig. 1:**
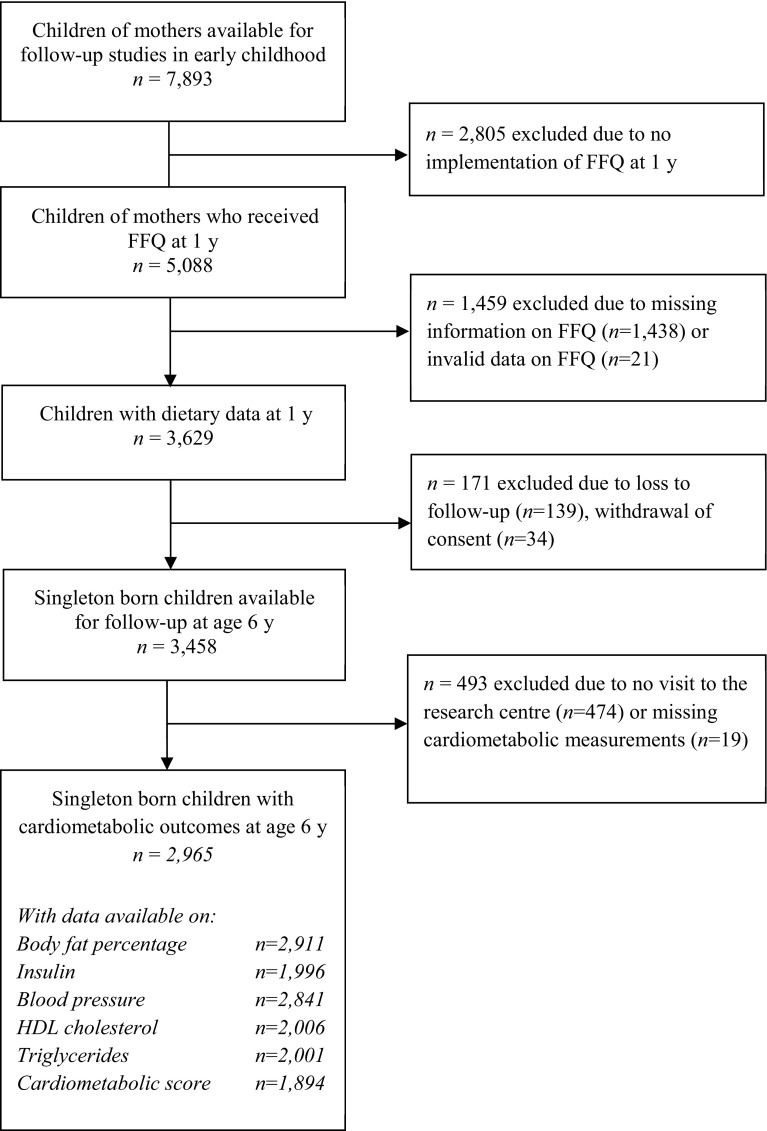
Flowchart of study participants included for the main analysis

This study was embedded in the Generation R Study, a population-based prospective cohort study from fetal life onward in Rotterdam, the Netherlands [18]. All parents provided written informed consent, and 7893 children were available for follow-up studies in early childhood [18]. A questionnaire on child diet was implemented at a later stage of the study and was sent to 5088 mothers who provided consent for follow-up and had sufficient mastery of the Dutch language. In total, 3650 (72 %) of these mothers returned the questionnaire, of whom 3629 provided valid dietary data [19]. Of this group, 2965 single-born children visited the research center at the age of 6 years and had one or more cardiometabolic measurements available (Fig. 1). Not all children had information available on every outcome, mainly because some parents or children did not give consent for blood collection [20]. The number of children included in this study therefore differs per outcome, ranging from 1894 for the cardiometabolic score to 2911 for body fat measurements (Fig. 1).

### Dietary assessment

Food intake was assessed at a median age of 12.9 months (95 % range 12.2–18.9) using a semiquantitative food frequency questionnaire (FFQ), covering the previous month [19]. This FFQ consisted of 211 food items that, according to a Dutch National Food Consumption Survey in 2002, are frequently consumed by children aged 9–18 months [21]. On the basis of standardized portion sizes and the Dutch Food Composition Table 2006 [22], food frequencies were converted into nutrient intakes. Evaluation of the FFQ against three 24-h recalls in a representative sample of 32 Dutch children with a median age of 14 months (95 % range 6–20) living in Rotterdam, showed an intraclass correlation coefficient of 0.7 for total protein intake [19, 23]. Mothers of a subgroup of 899 Dutch children [18] received an additional FFQ around their child’s age of 2 years (median 24.9 months, 95 % range 24.3–27.6) [23]. Of these children, 714 had cardiometabolic measurements available at the age of 6 years.

### Cardiometabolic outcome assessments

Children’s cardiometabolic health outcomes were measured at their median age of 5.9 years (95 % range 5.6–6.6) in our research center by well-trained staff [20]. Weight was determined with a mechanical personal scale (SECA, Almere, the Netherlands), and height was measured with a Harpenden stadiometer (Holtain Limited, Dyfed, UK). Body fat mass was measured using a dual-energy X-ray absorptiometry (DXA) scanner (iDXA, GE-Lunar, 2008, Madison, WI, USA) using enCORE software version 13.6. Body fat percentage (BF%) was calculated by expressing total fat mass as percentage of total body weight, and fat mass index (FMI) was calculated as total fat mass divided by height squared (kg/m^2^).

Non-fasting blood samples were obtained, transported, and stored as described in detail previously [20]. Serum concentrations of insulin, C-peptide, triglycerides, and total, HDL, and LDL cholesterol were measured at the Erasmus Medical Center with enzymatic methods (Cobas 8000, Roche, Almere, the Netherlands) [20]. Quality control samples demonstrated intra-assay coefficients of variation ranging from 0.77 to 1.17 % and inter-assay coefficients ranging from 0.87 to 1.69 %.

While the children were lying, systolic (SBP) and diastolic blood pressure (DBP) were measured at the right brachial artery for four times with 1-min intervals, using the validated automatic sphygmomanometer Datascope Accutorr PlusTM (Paramus, NJ, USA). We used mean SBP and mean DBP of the last three measurements. For all cardiometabolic outcomes, we calculated age- and sex-specific SD scores (SDS), on the basis of the total Generation R Study population with data available on cardiometabolic health at 6 years (*n* ranging from 4414 to 6491) [18].

In line with previous studies that defined scores for a metabolic syndrome-like phenotype in children [24], we created a continuous score including five components: BF%, blood pressure, HDL cholesterol (HDL-C), triglycerides, and insulin levels [24]. The cardiometabolic score was calculated as: BF%SDS + 0.5 × SBP SDS + 0.5 × DBP SDS + triglycerides SDS + (−1 × HDL−C SDS) + insulin SDS, with a higher score reflecting higher cardiometabolic risk.

### Covariates

Information on maternal age and educational level at enrollment was obtained with a questionnaire. Maternal height and weight were measured at our research center at enrollment in the study, and body mass index (BMI, kg/m^2^) was calculated [18]. Maternal smoking during pregnancy was assessed using questionnaires in each trimester and was categorized into never; quit in the first trimester; or continued. Information on child’s sex, birth weight, and gestational age was available from medical records and hospital registries, and gestational age- and sex-specific birth weight *Z*-scores were calculated [25]. Child’s ethnicity was defined as Dutch when both parents were born in the Netherlands and as non-Dutch when one or both parents were born in another country [26]. Information on breastfeeding (never, partial, or exclusive in the first 4 months) was obtained from delivery reports and postnatal questionnaires [19]. Child’s height and weight around the age of 1 year were measured at the Community Child Health Centers, and age- and sex-specific BMI-SDS were calculated using Dutch reference curves [27]. Child fat and carbohydrate intake was derived from the FFQs and energy adjusted using the residual method [28]. Child BMI (kg/m^2^) at 6 years was calculated from measured height and weight and expressed in age- and sex-specific SDS [27]. Child weight status (underweight, normal weight, or overweight) was defined using international age- and sex-specific BMI cutoffs [29]. Screen time and participation in sports (yes/no) at the age of 6 years were assessed with a questionnaire as proxies for physical activity level during early childhood.

### Statistical analysis

Because we were interested in the effect of protein independent of its energy content, we adjusted protein intake for total energy intake using the nutrient residual method [28]. To enhance interpretability, predicted protein intake for mean energy intake (1312 kcal/day) was added as constant. Protein intake was analyzed as continuous variable and categorized into tertiles. Insulin levels were square-root-transformed to obtain a normal distribution, and subsequently, age- and sex-specific SDS were created for all outcomes.

We used linear regression models to assess the associations of total, animal, and vegetable protein intake at the age of 1 year with cardiometabolic outcomes at the age of 6 years. Animal and vegetable protein intakes were adjusted for each other. Potential confounders were selected based on theory or previous literature. The following covariates were included in the multivariable models based on theory: child’s age, sex, ethnicity, birth weight *Z*-score, height for age, energy-adjusted fat intake, and total energy intake. Other potential confounders were entered individually in unadjusted models and were included in the final model only in case of a significant change (≥10 %) in the effect estimate of protein intake on at least one of the cardiometabolic outcomes [30]. Following this procedure, models were additionally adjusted for: maternal age, educational level, BMI, and smoking during pregnancy; and child’s breastfeeding, age at dietary measurement, screen time, and participation in sports. The following covariates were considered but not included because they did not fulfill the 10 % change criterion: household income, maternal parity, maternal alcohol consumption during pregnancy, child’s gestational age at birth, timing of introduction of solid foods, and food allergies.

For all outcomes except BF%, we included BF% at 6 years in a separate model to examine whether changes in body fat mediated the association with other cardiometabolic factors. To assess whether the associations between protein intake and cardiometabolic outcomes might differ by child sex, ethnicity, birth weight *Z*-score, or weight status at 6 years, we evaluated the statistical interaction by adding the product term of the potential effect modifier and total protein intake to the multivariable models.

We performed several sensitivity analyses. We additionally adjusted the multivariable models for child BMI-SDS at 1 year to assess whether protein intake at 1 year predicted childhood cardiometabolic health independent of baseline BMI. To examine whether associations with blood lipids, BP, or insulin levels could be explained by differences in body fat, we additionally adjusted these models for child BF% at 6 years in separate models. We performed macronutrient substitution analyses in which we adjusted our models for carbohydrate intake or for saturated, monounsaturated, and polyunsaturated fat intake, instead of total fat intake, to check whether it made a difference whether protein was consumed at the expense of different other macronutrients. To explore whether associations with the cardiometabolic score were driven by one component only, we performed sensitivity analyses in which we excluded one component of the score at a time. Because the FFQ was developed and validated for Dutch children, we repeated the analyses in Dutch children only. To explore potential selection bias due to missing blood sampling, we examined descriptive statistics and associations of protein intake with body fat and blood pressure measurements in children who had blood samples available (*n* = 2010). In the subgroup of children with dietary data at 2 years, we assessed associations of protein intake at this age with cardiometabolic outcomes at 6 years using the same multivariable linear regression models as for protein intake at the age of 1 year.

Missing values of covariates were multiple imputed (*n* = 10 imputations) according to the fully conditional specification method (predictive mean matching) [31]. We report the pooled regression coefficients after the multiple imputation procedure. Statistical analyses were performed using SPSS Statistics version 21.0 (IBM Corp., Armonk, NY, USA).

## Results

### Subject characteristics

Characteristics of the children and their mothers are presented in Table 1. Mean (±SD) total protein intake at the age of 1 year was 41.2 g (±12.9), corresponding to 12.9 % (±2.4) of total energy intake (*E*%). This is higher than recommended for this age group, but similar to intakes observed in the general Dutch and other Western pediatric populations [32, 33]. Boys had a higher total energy and absolute protein intake than girls, but *E*% from protein was not different. Energy-adjusted protein intake was also similar among children with and without blood samples (Supplemental Table S1).

**Table 1:** Characteristics of the participating children and their mothers

	All (*n* = 2965)	Boys (*n* = 1445)	Girls (*n* = 1520)
Maternal characteristics			
Maternal age (year)	31.9 (21.8–39.8)	31.9 (21.7–40.0)	31.9 (21.9–39.6)
Maternal BMI at enrollment (kg/m^2^)	23.7 (19.0–35.2)	23.4 (18.7–35.2)	23.6 (19.0–36.0)
Education level (%)			
Primary	5.2	5.2	5.1
Secondary	36.3	34.9	37.6
Higher	58.5	59.9	57.3
Smoking during pregnancy (%)			
Never	78.1	78.3	78.6
Until pregnancy was known	10.3	8.9	10.9
Continued	11.6	12.8	10.5
Child characteristics			
Girls (%)	51.3	–	–
Dutch ethnicity (%)	68.8	69.5	68.4
Gestational age at birth (wk)	39.9 (1.8)	39.9 (1.8)	40.1 (1.8)
Birth weight (g)	3452 (569)	3524 (576)	3383 (555)
Breastfeeding (%)			
Exclusive in the first 4 months	31.4	31.8	31.0
Partial in the first 4 months	60.7	60.6	60.9
Never	7.9	7.6	8.1
Child characteristics at dietary measurement			
Age at FFQ (mo)	12.9 (12.2–18.9)	12.9 (12.2–19.1)	12.9 (12.2–18.8)
Total energy intake (kcal/day)	1265 (678–2206)	1316 (691–2210)	1221 (652–2230)
Protein intake (g/day)			
Total protein	41.8 (12.6)	42.9 (13.0)	40.6 (12.2)
Animal protein	25.6 (10.2)	26.9 (10.5)	25.7 (9.8)
Vegetable protein	14.2 (5.6)	15.5 (5.5)	14.4 (5.7)
Protein intake (*E*%)			
Total protein	12.9 (2.4)	12.9 (2.4)	12.9 (2.4)
Animal protein	8.1 (2.4)	8.1 (2.4)	8.2 (2.4)
Vegetable protein	4.7 (1.4)	4.7 (1.3)	4.6 (1.5)
Child characteristics at 6-year visit			
Age (year)	5.9 (5.7–6.6)	5.9 (5.6–6.6)	5.9 (5.6–6.5)
Screen time (h/day)	1.3 (0.3–4.3)	1.3 (0.3–4.8)	1.2 (0.2–4.6)
Participation in sports (%)	44.2	43.0	45.5
Height (cm)	118.2 (5.2)	118.5 (5.1)	117.9 (5.2)
Weight (kg)	22.4 (3.4)	22.5 (3.4)	21.7 (3.4)
BMI (kg/m^2^)	16.0 (1.6)	16.0 (1.6)	16.0 (1.7)
Body fat percentage (%)	23.5 (16.2–36.4)	21.1 (15.7–33.5)	25.6 (18.8–37.5)
Systolic blood pressure (mmHg)	102 (8)	101 (8)	102 (8)
Diastolic blood pressure (mmHg)	60 (7)	60 (7)	61 (6)
HDL cholesterol (mmol/L)	1.35 (0.31)	1.36 (0.31)	1.33 (0.30)
Triglyceride levels (mmol/L)	0.97 (0.40–2.36)	0.96 (0.38–2.34)	0.98 (0.44–2.47)
Insulin levels (pmol/L)	115 (18–398)	119 (17–382)	115 (19–432)

Values are percentages for categorical variables, means (SD) for continuous variables with a normal distribution, or medians (95 % range) for continuous variables with a skewed distribution

*E%* energy percentage, *FFQ* food frequency questionnaire

### Associations between total protein intake and cardiometabolic outcomes

After adjustment for covariates, children with a protein intake in the highest tertile had a 0.08 SD (95 % CI 0.01; 0.16) higher BF%, 0.14 SD (95 % CI −0.24; −0.03) lower triglyceride levels, and a 0.09 SD (95 % CI −0.18; 0.00) lower DBP than children in the lowest tertile of protein intake (Table 2). Protein intake was not significantly associated with SBP, insulin, HDL-C, or the cardiometabolic score.

**Table 2:** Covariate-adjusted associations of total protein intake at the age of 1 year with cardiometabolic outcomes at the age of 6 years

	BF % (SDS)	Insulin (SDS)	SBP (SDS)	DBP (SDS)	HDL-C (SDS)	Triglycerides (SDS)	Cardiometabolic risk factor score
Whole group	*n* = 2911	*n* = 1996	*n* = 2841	*n* = 2841	*n* = 2006	*n* = 2001	*n* = 1894
Protein intake^a^ per 10 g/day	**0.04** (**0.00, 0.08**)	0.02 (−0.03, 0.08)	0.00 (−0.05, 0.05)	−0.04 (−0.09, 0.01)	0.03 (−0.03, 0.09)	−**0.07** (−**0.13,** −**0.01**)	−0.09 (−0.24, 0.05)
Tertile 1	Reference	Reference	Reference	Reference	Reference	Reference	Reference
Tertile 2	0.03 (−0.04, 0.11)	0.04 (−0.07, 0.15)	−0.08 (−0.17, 0.01)	−0.02 (−0.12, 0.06)	0.05 (−0.05, 0.16)	−0.08 (−0.19, 0.03)	−0.12 (−0.39, 0.15)
Tertile 3	**0.08** (**0.01, 0.16**)	0.01 (−0.10, −0.12)	−0.05 (−0.13, 0.04)	−**0.09** (−**0.18,** −**0.00**)	0.05 (−0.06, 0.16)	−**0.14** (−**0.24,** −**0.03**)	−0.17 (−0.44, 0.10)
*P* for trend^b^	**0.03**	0.22	0.32	**0.04**	0.35	**0.01**	0.21
Girls	*n* = 1487	*n* = 980	*n* = 1426	*n* = 1457	*n* = 1457	*n* = 984	*n* = 982
Protein intake^a^ per 10 g/day	**0.07** (**0.02, 0.13**)	**0.10** (**0.01, 0.19**)	0.00 (−0.07, 0.07)	−0.03 (−0.10, 0.04)	0.00 (−0.09, 0.09)	−0.01 (−0.10, 0.08)	0.05 (−0.17, 0.27)
Tertile 1	Reference	Reference	Reference	Reference	Reference	Reference	Reference
Tertile 2	0.10 (−0.01, 0.20)	0.05 (−0.06, 0.25)	−0.05 (−0.17, 0.07)	0.03 (−0.09, 0.15)	0.02 (−0.13, 0.17)	0.00 (−0.15, 0.15)	0.10 (−0.27 0.48
Tertile 3	**0.11** (**0.01, 0.21**)	**0.15** (**0.00, 0.31**)	−0.09 (−0.22, 0.03)	−0.10 (−0.22, 0.03)	0.02 (−0.14, 0.17)	−0.02 (−0.17, 0.14)	0.04 (−0.35, 0.43)
*P* for trend^b^	**0.03**	**<0.05**	0.14	0.20	0.83	0.85	0.84
Boys	*n* = 1422	*n* = 1016	*n* = 1381	*n* = 1384	*n* = 1384	*n* = 1017	*n* = 1013
Protein intake^a^ per 10 g/day	0.01 (−0.05, 0.07)	−0.05 (−0.13, 0.03)	−0.01 (−0.08, 0.06)	−0.04 (−0.11, 0.03)	0.05 (−0.03, 0.13)	−**0.12** (−**0.20,** −***0.04***)	−**0.22** (−**0.42, 0.02**)
Tertile 1	Reference	Reference	Reference	Reference	Reference	Reference	Reference
Tertile 2	−0.05 (−0.16, 0.06)	0.03 (−0.13, 0.17)	0.11 (−0.23, 0.01)	−0.08 (−0.21, 0.05)	0.06 (−0.10, 0.21)	−**0.16** (−**0.31,** −**0.01**)	−0.36 (−0.74, 0.03)
Tertile 3	0.04 (−0.07, 0.14)	−0.11 (−0.26, 0.03)	−0.03 (−0.15, 0.10)	−**0.12** (−**0.23, 0.00**)	0.11 (−0.04, 0.27)	−**0.25** (−**0.40,** −**0.10**)	−**0.40** (−**0.77,** −**0.02**)
*P* for trend^b^	0.50	0.11	0.68	0.06	0.14	**<0.01**	**0.02**

Values are based on multivariable linear regression models and reflect differences (95 % CI) in individual cardiometabolic outcomes (age- and sex-adjusted SDS) and in cardiometabolic risk factor score per 10 g/day increase in protein intake, and for tertiles of protein intake, as compared to the lowest tertile

*p* values for interaction between total protein intake and child sex were: 0.05 for BF%; 0.01 for insulin; 0.79 for SBP; 0.58 for DBP; 0.08 for HDL-C; <0.01 for triglycerides; and 0.07 for the cardiometabolic score

Models are adjusted for maternal age, BMI, education, and smoking during pregnancy; and child’s ethnicity, birth weight *Z*-score, breastfeeding in the first 4 months of life, age at dietary measurement, total energy intake, energy-adjusted fat intake, height SDS at 6 year, participation in sports at 6 year, and screen time at 6 year. Significant effect estimates are indicated in **bold**

*SDS* standard deviation score, *BF%* body fat percentage, *SBP* systolic blood pressure, *DBP* diastolic blood pressure, *HDL-C* high-density lipoprotein cholesterol

^a^Protein intakes are energy adjusted using the residual method. Tertiles are computed based on the total population for analysis (Fig. 1, *n* = 2965), and the distribution was similar in boys and girls. Mean protein intake in the tertiles was 34.5, 41.7, and 50.2 g/day

^b^Tests for trend were conducted using the tertiles of protein intake as a continuous variable

Because we observed significant or borderline significant interactions between total protein intake and sex on BF% (*p* = 0.05), insulin (*p* = 0.01), C-peptide (*p* = 0.03), HDL-C (*p* = 0.08), triglycerides (*p* < 0.01), and the cardiometabolic score (*p* = 0.07), we also performed all analyses in boy and girls separately (Table 2). These stratified analyses revealed that higher protein intake was associated with a higher BF% and higher insulin levels in girls, but not in boys. In contrast, higher protein intake was associated with lower triglyceride levels in boys, but not in girls. The association between protein intake and DBP was slightly stronger in boys than in girls, but in both groups, effect estimates were similar to those observed in the whole population. Finally, protein intake was not associated with the cardiometabolic score in girls, while in boys, higher intake was associated with a lower cardiometabolic risk factor score [−0.56 SD (95 % CI −0.92; −0.20) per 10 g/day of total protein intake].

Additional adjustment for BMI at 1 year (data not shown) or BF% at 6 years (Supplemental Table S2) did not change the results. Associations of protein intake with C-peptide levels were similar to those observed with insulin levels, results for FMI were similar to those observed for BF%, and no associations were observed with total or LDL cholesterol (Supplemental Table S3). Results from unadjusted models were similar to those from the adjusted models, with similar differences between boys and girls (Supplemental Table S4).

### Additional analyses

The association between protein intake and BF% in girls was mainly driven by animal protein intake, whereas effect estimates for other outcomes were similar for animal and vegetable protein intake (Supplemental Table S5). Associations between protein intake and cardiometabolic outcomes did not significantly differ by the children’s ethnicity, weight status at 6 years, or birth weight *Z*-score. Adjusting the models for total carbohydrate or different fatty acids instead of total fat intake rendered similar results for protein intake (data not shown). Sensitivity analyses in Dutch children only (*n* = 1965) showed similar effect estimates as compared to the whole group, except for DBP for which the effect estimates were slightly larger in Dutch children (Supplemental Table S6). Sensitivity analyses restricted to children with blood sample available rendered similar effect estimates for body fat and blood pressure (data not shown). Analyses in which we excluded one component from the cardiometabolic score at a time revealed similar associations. In the subgroup of children with dietary data at age 2 years (*n* = 714), protein intake at the age of 2 years was no longer associated with BF% or insulin levels at 6 years in girls, while in boys, it was associated with a lower BF% (Supplemental Table S7). Associations between protein intake at age 2 years and other cardiometabolic outcomes were similar to those observed for protein intake at age 1 year, but with wider confidence intervals.

## Discussion

This large prospective population-based study suggests that protein intake at the age of 1 year is associated with cardiometabolic health at school age, but that these associations differ by sex. A higher protein intake was associated with a higher BF% and higher insulin levels in girls and with lower triglyceride levels, lower DBP, and a lower cardiometabolic risk factor score in boys. Protein intake was not consistently associated with SBP or HDL cholesterol levels at the age of 6 years. The associations with BP, insulin, and triglyceride levels were independent of BF%. Although the effect estimates were small and may not have direct consequences on an individual level, they remained statistically significant after adjustment for several confounders and may be relevant on a population level in predicting later cardiometabolic disease risk [2, 3].

### Interpretation and comparison with previous studies

The association between protein intake in early life and later obesity is in line with results from a large European trial in 1090 infants. In this study, children who received high-protein formula in infancy had a higher BMI at 6 years than children who received lower-protein formula [12]. However, only a few previous studies examined associations between protein intake in the first years of life and later measures of body fat. Two observational studies reported no association between early-life protein intake and BF% at the ages of 4 or 10 years [34, 35], whereas two other studies reported positive associations with skinfold thicknesses at 7 or 8 years [9, 10].

Nevertheless, none of these studies reported differences between boys and girls. The observed differences between boys and girls in our study might be explained by a difference in timing of adiposity rebound (AR) or in peak BMI at AR. The AR corresponds to a rise in BMI curve followed by a rise in fat mass index that occurs between the age of 5 and 7 years [36, 37] and tends to occur earlier in girls than in boys [38]. A previous study reported that higher protein intake in early childhood is associated with an earlier AR [10], whereas in other studies, no consistent relation was observed with timing of AR [39, 40]. One of these latter studies, however, did observe that a higher protein intake in early childhood was associated with a higher BMI at AR in girls, but not in boys [40].

Another potential mechanism for the observed differences between boys and girls is a difference in endocrine response to high protein intake [41]. High protein intake has been associated with increased IGF-1 secretion, which may mediate the relation between protein intake and obesity [41]. In the previously mentioned European trial with high- and low-protein infant formulas, although no sex differences were observed in the association between the intervention and growth [12], girls had a stronger IGF-1 response to the higher protein formula than boys [41]. In line with these findings, we observed clear sex differences in the association between protein intake and insulin levels, which also acts as a growth hormone [41]. Protein intake was strongly associated with higher insulin levels in girls, but not at all in boys. When we adjusted the BF% models in girls for insulin levels, the association between protein intake and BF% was attenuated, which suggests that insulin might mediate the association with BF%. Studies in adults show that dietary proteins stimulate the secretion of insulin [42], and high protein intake has been related to an increased risk of type 2 diabetes [43]. However, previous studies in children did not report effects of high protein intake on measures of insulin sensitivity [17, 44, 45].

We observed that higher protein intake was associated with a lower DBP, but not consistently with SBP. A previous study reported inverse associations between protein intake and both SBP and DBP in 2.5-year-old children [46], while two other studies observed no associations [47, 48]. In line with our findings, a large observational study in 1605 adolescents (12.5–17.5 years) showed an inverse association between protein intake and DBP, but not SBP in boys [49]. Several meta-analyses of studies in adults also report inverse associations between protein intake and blood pressure [5–7]. The mechanisms underlying a beneficial effect of protein intake on blood pressure have not yet been clarified and may differ for intake of different amino acids [49]. Proposed pathways of a blood-pressure-lowering effect of protein include increased synthesis of ion channels; increased renal plasma flow; increased glomerular filtration rate; or vasodilating effects of certain amino acids [5, 7]. However, we have previously shown in our population that protein intake in early childhood is not associated with glomerular filtration rate [50].

The present study shows that protein intake was not associated with total, HDL or LDL cholesterol levels, but we did observe lower triglyceride levels in relation to higher protein intake in boys. These latter findings correspond to results from a previous study in adolescents, in which higher absolute protein intake was associated with lower triglyceride and cholesterol levels [45]. However, in this study, the association was no longer significant after adjustment for total energy intake. Other previous studies in children showed no association between protein intake and cholesterol or triglyceride levels (reviewed by [17]). However, in line with our results, a meta-analysis of trials in adults showed that subjects consuming a higher protein diet had lower triglyceride levels than subjects with a lower-protein diet [6]. Potential mechanisms of a direct effect of protein intake on lipid profile are unknown. A high carbohydrate diet, which could correspond to a low-protein diet, has been shown to reduce triglyceride clearance and thereby increase serum levels of triglycerides [51]. In our analyses, however, effect estimates for protein intake were similar after adjusting for either carbohydrate or fat intake.

In our study, we observed differences in animal versus vegetable protein intake on the association with body fat, but not on the other cardiometabolic outcomes. In one previous study, it was also reported that animal, and more specifically, dairy protein, but not meat or cereal protein intake at 1 year was associated with child body fat at 7 years [16]. Animal and vegetable protein might have different effects on adiposity via differences in IGF-1: Another study reported that intake of animal, but not vegetable protein in 2.5-year-old children was associated with higher IGF-1 concentrations [15]. Studies in adults report inconsistent results for differences in effects of animal and vegetable protein on cardiometabolic diseases [43, 52]. Further studies, both in adult and in child populations, are needed to elucidate the effects of animal and vegetable protein on cardiometabolic health.

### Methodological considerations

Important strengths of this study are its prospective population-based design and the large number of subjects being studied. Of all mothers who received the FFQ, 72 % returned the questionnaire. These mothers were generally higher educated and had a more healthy lifestyle than mothers who did not return the FFQ [23]. Of all children of whom information was available on food intake, more than 80 % participated in the follow-up measurements at 6 years. Blood samples were available in 67 % of these children, who had on average higher educated mothers than children without blood samples, but were not different with regard to protein intake, body fat, or blood pressure (Supplemental Table S1). Furthermore, associations between protein intake and body fat or blood pressure were not different among the children with blood samples available than among the whole group.

The FFQ was sent to Dutch-speaking mothers only, but with different ethnic backgrounds. A limitation of our FFQ is that it was only validated for Dutch children [19]. However, sensitivity analyses restricted to Dutch children revealed similar results, suggesting that in our analyses, no large bias due to ethnicity was present. Strengths of our dietary assessment are that an FFQ measures habitual diet rather than dietary intake at just one or a few days, and that we calculated not only total protein intake, but also broken down in protein from animal and vegetable sources. A limitation is that we did not have dietary data at 6 years and therefore could not study whether the associations of early diet were independent of current diet. Because we adjusted our models for total energy and fat intake, the effect estimates can be interpreted as the effect of exchanging carbohydrate for a similar number of calories from protein. Adjusting the models for other macronutrients rendered similar results, suggesting that for cardiometabolic outcomes, it does not matter whether fat or carbohydrate is exchanged for protein in the diets of young children.

An important strength of our study is that we had information on many potential parental and child confounders for which we adjusted in our analyses. However, because of the observational design of our study, residual confounding of other lifestyle-related variables, such as physical activity, may still be present. We used screen time and participation in sports at the age of 6 years as proxies for physical activity during early childhood, but unfortunately, we had no information available on physical activity of the children at earlier ages.

Finally, we performed detailed measurements of childhood adiposity and cardiometabolic health. Because we evaluated multiple outcomes, this might have increased the risk of chance findings (type I errors) due to multiple testing. Nevertheless, because the cardiometabolic outcomes considered are correlated, we did not adjust for multiple comparisons. In addition, we combined the individual risk factors in a continuous cardiometabolic score. Advantages over a dichotomous metabolic syndrome definition are that a continuous score is less prone to error and more sensitive to pick up differences because more information is used [24].

## Conclusion

In this prospective cohort study in young children with high protein intake, we observed differences in associations of protein intake with cardiometabolic outcomes between boys and girls. Protein intake in early childhood was associated with higher insulin levels and a higher BF% at 6 years in girls and with lower triglyceride levels and a lower cardiometabolic risk factor score in boys. In both sexes, protein intake tended to be associated with a lower diastolic blood pressure. Further studies are needed to explore whether and how protein intake differently affects cardiometabolic health in boys and girls and to investigate whether the observed changes in cardiometabolic outcomes in childhood persist into adulthood.

## Electronic supplementary material

Below is the link to the electronic supplementary material.

Supplementary material 1 (PDF 188 kb)
