# Supplementary material for: Protein intake in early childhood and cardiometabolic health at school age: the Generation R Study
Source: Eur J Nutr. 2015 Sep 2;55(6):2117–27. doi: 10.1007/s00394-015-1026-7 (PMC5009172; doi:10.1007/s00394-015-1026-7)
Supplement: Supplementary file 1 — Supplementary material 1 (PDF 188 kb) [file 394_2015_1026_MOESM1_ESM.pdf]

Supplemental Table S1. Characteristics of subjects with and without child blood samples.

	With blood samples (*n* = 2,006)	Without blood samples (*n* = 959)
**Maternal characteristics**		
Maternal age (y)	31.9 (22.1-39.9)	31.7 (21.0-39.6)
Maternal BMI at enrollment (kg/m^2)	23.4 (18.9-35.2)	23.6 (18.8-36.0)
Education level (%)		
- Primary	5.6	6.2
- Secondary	35.0	36.7
- Higher	59.4	57.1
Smoking during pregnancy (%)		
- Never	78.5	78.3
- Until pregnancy was known	10.0	9.7
- Continued	11.5	12.0
**Child characteristics**		
Girls (%)	48.8	55.2
Dutch ethnicity (%)	69.2	68.1
Gestational age at birth (wk)	39.9 (1.7)	40.0 (1.8)
Birth weight (g)	3482 (550)	3453 (552)
Breastfeeding (%)		
- Exclusive in the first 4 months	29.2	27.7
- Partial in the first 4 months	61.7	61.6
- Never	9.1	10.7
**Child characteristics at dietary measurement**		
Age at FFQ (mo)	12.9 (12.2-19.0)	12.9 (12.2-18.8)
Total energy intake (kcal/d)	1283 (664-2164)	1245 (691-2279)
Protein intake (g/d)		
- Total protein	41.4 (12.7)	40.8 (13.1)
- Animal protein	25.9 (10.1)	25.3 (10.6)
- Vegetable protein	15.0 (5.6)	14.9 (5.8)
Protein intake (E%)		
- Total protein	12.9 (2.4)	12.8 (2.4)
- Animal protein	8.1 (2.4)	8.0 (2.5)
- Vegetable protein	4.6 (1.4)	4.7 (1.4)
**Child characteristics at 6 y visit**		
Age (y)	5.9 (5.7-6.6)	5.9 (5.6-6.5)
Screen time (h/d)	1.3 (0.2-4.4)	1.3 (0.2-4.2)
Participation in sports (%)	43.9	43.0
Height (cm)	118.4 (5.1)	117.8 (5.3)
Weight (kg)	22.5 (3.3)	22.3 (3.5)
BMI (kg/m^2)	16.0 (1.6)	16.0 (1.7)
Body fat percentage (%)	23.3 (16.2-36.1)	24.1 (16.3-37.5)
Systolic blood pressure (mmHg)	102 (8)	103 (8)
Diastolic blood pressure (mmHg)	60 (6)	61 (7)

Values are percentages for categorical variables, means (SD) for continuous variables with a normal distribution, or medians (95% range) for continuous variables with a skewed distribution.

Abbreviations: BMI, body mass index; E%, energy percentage; FFQ, food frequency questionnaire.

Supplemental Table S2. Covariate-adjusted associations of total protein intake at the age of 1 year with cardiometabolic outcomes at 6 years, additionally adjusted for body fat percentage.

	Insulin (SDS)	SBP (SDS)	DBP (SDS)	HDL-C (SDS)	Triglycerides (SDS)	Cardiometabolic risk factor score
**Whole group**	*n* = 1,996	*n* = 2,841	*n* = 2,841	*n* = 2,006	*n* = 2,001	*n* = 1,894
Per 10 g/d	*0.02 (-0.04, 0.08)*	*-0.01 (-0.06, 0.04)*	*-0.04 (-0.09, 0.00)*	*0.03 (-0.03, 0.08)*	***-0.07 (-0.13, -0.01)***	*-0.12 (-0.25, 0.01)*
Tertile 1	Reference	Reference	Reference	Reference	Reference	Reference
Tertile 2	0.04 (-0.07, 0.15)	-0.08 (-0.17, 0.01)	-0.03 (-0.11, 0.06)	0.05 (-0.05, 0.16)	0.08 (-0.19, 0.03)	-0.13 (-0.37, 0.11)
Tertile 3	0.01 (-0.10, 0.11)	-0.06 (-0.15, 0.03)	**-0.10 (-0.18, -0.01)**	0.05 (-0.05, 0.16)	**-0.14 (-0.25, -0.03)**	**-0.25 (-0.50, -0.01)**
P for trend[#]	0.91	0.22	**0.03**	0.33	**0.01**	**0.04**
**Girls**	*n* = 980	*n* = 1,426	*n* = 1,457	*n* = 1,457	*n* = 984	*n* = 982
Per 10 g/d	***0.10 (0.01, 0.19)***	*-0.01 (-0.08, 0.07)*	*-0.04 (-0.11, 0.03)*	*0.01 (-0.07, 0.09)*	*-0.01 (-0.10, 0.08)*	0.02 (-0.17, 0.23)
Tertile 1	Reference	Reference	Reference	Reference	Reference	Reference
Tertile 2	0.09 (-0.06, 0.24)	-0.05 (-0.17, 0.07)	0.02 (-0.10, 0.14)	0.02 (-0.13, 0.17)	0.02 (-0.14, 0.17)	0.03 (-0.33 0.39)
Tertile 3	0.13 (-0.02, 0.29)	-0.08 (-0.21, 0.04)	-0.08 (-0.20, 0.05)	0.02 (-0.14, 0.17)	0.02 (-0.14, 0.17)	0.03 (-0.34, 0.40)
P for trend[#]	0.10	0.19	0.23	0.99	0.88	0.99
**Boys**	*n* = 1,422	*n* = 1,016	*n* = 1,381	*n* = 1,384	*n* = 1,384	*n* = 1,017
Per 10 g/d	*-0.05 (-0.13, 0.03)*	*-0.01 (-0.08, 0.06)*	*-0.05 (-0.12, 0.01)*	*0.06 (-0.01, 0.14)*	***-0.12 (-0.21, -0.04)***	***-0.24 (-0.42, -0.05)***
Tertile 1	Reference	Reference	Reference	Reference	Reference	Reference
Tertile 2	0.05 (-0.10, 0.21)	-0.12 (-0.24, 0.01)	-0.06 (-0.19, 0.07)	0.06 (-0.10, 0.21)	-0.08 (-0.23, 0.08)	-0.22 (-0.58, 0.14)
Tertile 3	-0.12 (-0.27, 0.04)	-0.03 (-0.16, 0.09)	**-0.13 (-0.25, 0.00)**	0.11 (-0.04, 0.27)	**-0.26 (-0.41, -0.11)**	**-0.57 (-0.92, -0.21)**
P for trend[#]	0.13	0.60	**0.05**	0.16	**<0.01**	**<0.01**

Values are based on multivariable linear regression models and reflect differences (95%CI) in individual cardiometabolic outcomes (age and sex adjusted SD scores) and in cardiometabolic score score per 10 g/d increase in protein intake, and for tertiles of protein intake, as compared to the lowest tertile. Significant effect estimates are indicated in ***bold****.*

Protein intakes are energy-adjusted using the residual method. Tertiles are computed based on the total population for analysis (Figure 1, n=2,965).

Models are adjusted for maternal age, BMI, education, and smoking during pregnancy; and child's ethnicity, birth weight Z-score, breastfeeding in the first four months of life, age at dietary measurement, energy intake, fat intake, height-for-age at 6y, participation in sports at 6y, and screen time at 6y.

[#] *Tests for trend were conducted using the tertiles of protein intake as a continuous variable.*

Abbreviations: SDS, standard deviation score; BF%, body fat percentage; SBP, systolic blood pressure; DBP, diastolic blood pressure; HDL-C, high-density lipoprotein cholesterol.

Supplemental Table S3. Covariate-adjusted associations of total protein intake at the age of 1 year with secondary cardiometabolic outcomes at 6 years.

	FMI (SDS)	C-peptide (SDS)	Total cholesterol (SDS)	LDL cholesterol (SDS)
**Whole group**	*n = 2,909*	*n* = 1,996	*n* = 2,006	*n* = 2,006
Per 10 g/d	***0.04 (0.00, 0.08)***	*0.04 (-0.02, 0.9)*	*-0.01 (-0.07, 0.05)*	*-0.01 (-0.07, 0.05)*
Tertile 1	Reference	Reference	Reference	Reference
Tertile 2	0.05 (-0.02, 0.12)	0.00 (-0.11, 0.11)	0.07 (-0.04, 0.17)	0.04 (-0.07, 0.15)
Tertile 3	**0.08 (0.01, 0.15)**	0.00 (-0.11, 0.11)	0.05 (-0.06, 0.16)	0.05 (-0.05, 0.16)
P for trend[#]	**0.03**	0.97	0.37	0.33
**Girls**		*n* = 980	*n* = 1,457	*n* = 1,457
Per 10 g/d	***0.06 (0.00, 0.11)***	***0.10 (0.02, 0.18)***	*0.01 (-0.07, 0.08)*	*-0.01 (-0.09, 0.07)*
Tertile 1	Reference	Reference	Reference	Reference
Tertile 2	**0.11 (0.02, 0.20)**	0.06 (-0.09, 0.22)	0.09 (-0.06, 0.24)	0.07 (-0.08, 0.22)
Tertile 3	**0.10 (0.00, 0.19)**	0.10 (-0.05, 0.26)	0.03 (-0.12, 0.18)	0.00 (-0.15, 0.15)
P for trend[#]	**0.04**	0.08	0.68	0.98
**Boys**		*n* = 1,016	*n* = 1,384	*n* = 1,384
Per 10 g/d	*0.02 (-0.03, 0.07)*	*-0.02 (-0.09, 0.06)*	*-0.01 (-0.08, 0.07)*	*-0.03 (-0.10, 0.05)*
Tertile 1	Reference	Reference	Reference	Reference
Tertile 2	-0.02 (-0.12, 0.08)	-0.07 (-0.22, 0.08)	-0.06 (-0.21, 0.09)	-0.09 (-0.24, 0.07)
Tertile 3	0.04 (-0.06, 0.14)	-0.12 (-0.27, 0.03)	0.02 (-0.14, 0.17)	0.01 (-0.15, 0.16)
P for trend[#]	0.45	0.20	0.83	0.92

Values are based on multivariable linear regression models and reflect differences (95%CI) in C-peptide and cholesterol levels (age and sex adjusted SD scores) per 10 g/d increase in protein intake, and for tertiles of protein intake, as compared to the lowest tertile. Significant effect estimates are indicated in ***bold****.*

Protein intakes are energy-adjusted using the residual method. Tertiles are computed based on the total population for analysis (Figure 1, n=2,965).

Models are adjusted for maternal age, BMI, education, and smoking during pregnancy; and child's ethnicity, birth weight Z-score, breastfeeding in the first four months of life, age at dietary measurement, energy intake, fat intake, height-for-age at 6y, weight-for-age at 6y, participation in sports at 6y, and screen time at 6y.

[#] *Tests for trend were conducted using the tertiles of protein intake as a continuous variable.*

Abbreviations: SDS, standard deviation score.

Supplemental Table S4. Crude associations of total protein intake at the age of 1 year with cardiometabolic outcomes at 6 years.

	BF% (SDS)	Insulin (SDS)	SBP (SDS)	DBP (SDS)	HDL-C (SDS)	Triglycerides (SDS)	Cardiometabolic risk factor score
**Whole group**	*n* = 2,909	*n* = 1,996	*n* = 2,841	*n* = 2,841	*n* = 2,006	*n* = 2,001	*n* = 1,894
Per 10 g/d	***0.06 (0.02, 0.11)***	*0.03 (-0.03, 0.09)*	*0.01 (-0.04, 0.06)*	*-0.03 (-0.08, 0.02)*	*0.02 (-0.04, 0.08)*	***-0.07 (-0.13, -0.01)***	-0.05 (-0.20, 0.11)
Tertile 1	Reference	Reference	Reference	Reference	Reference	Reference	Reference
Tertile 2	0.03 (-0.05, 0.11)	0.05 (-0.06, 0.15)	-0.07 (-0.16, 0.02)	-0.03 (-0.11, 0.06)	0.05 (-0.06, 0.15)	-0.07 (-0.18, 0.03)	-0.12 (-0.41, 0.16)
Tertile 3	**0.11 (0.03, 0.19)**	0.03 (-0.09, 0.13)	-0.02 (-0.11, 0.07)	**-0.08 (-0.17, -0.00)**	0.03 (-0.07, 0.14)	**-0.14 (-0.25, -0.03)**	-0.09 (-0.36, 0.18)
P for trend[#]	**<0.01**	0.69	0.70	0.11	0.49	**<0.01**	0.51
**Girls**	*n* = 1,487	*n* = 980	*n* = 1,426	*n* = 1,457	*n* = 1,457	*n* = 984	*n* = 982
Per 10 g/d	***0.09 (0.03, 0.15)***	***0.11 (0.02, 0.20)***	*0.02 (-0.05, 0.09)*	*-0.03 (-0.10, 0.04)*	*-0.01 (-0.10,0.08)*	*-0.02 (-0.11, 0.07)*	*0.09 (-0.14, 0.31)*
Tertile 1	Reference	Reference	Reference	Reference	Reference	Reference	Reference
Tertile 2	0.08 (-0.03, 0.20)	0.10 (-0.05, 0.26)	-0.05 (-0.17, 0.07)	0.00 (-0.12, 0.11)	0.02 (-0.14, 0.16)	0.02 (-0.13, 0.18)	0.07 (-0.33, 0.46)
Tertile 3	**0.12 (0.01, 0.23)**	**0.16 (0.00, 0.31)**	-0.06 (-0.19, 0.07)	-0.08 (-0.21, 0.04)	-0.01 (-0.16, 0.15)	0.00 (-0.16, 0.15)	0.13 (-0.28, 0.54)
P for trend[#]	**0.03**	**0.04**	0.35	0.22	0.94	0.99	0.52
**Boys**	*n* = 1,422	*n* = 1,016	*n* = 1,381	*n* = 1,384	*n* = 1,384	*n* = 1,017	*n* = 1,013
Per 10 g/d	*0.04 (-0.03, 0.10)*	*-0.03 (-0.12, 0.05)*	*0.01 (-0.06, 0.08)*	*-0.03 (-0.10, 0.04)*	*0.05 (-0.03, 0.13)*	***-0.12 (-0.20, -0.04)***	*-0.15 (-0.35, 0.05)*
Tertile 1	Reference	Reference	Reference	Reference	Reference	Reference	Reference
Tertile 2	-0.03 (-0.15, 0.09)	0.08 (-0.07, 0.23)	-0.09 (-0.21, 0.04)	-0.05 (-0.18, 0.08)	0.04 (-0.11, 0.20)	-0.08 (-0.23, 0.07)	-0.15 (-0.54, 0.24)
Tertile 3	0.04 (-0.06, 0.17)	-0.09 (-0.24, 0.06)	0.03 (-0.10, 0.15)	-0.09 (-0.21, 0.04)	0.08 (-0.07, 0.23)	**-0.24 (-0.39, -0.09)**	-0.35 (-0.74, 0.03)
P for trend[#]	0.38	0.21	0.68	0.19	0.29	**<0.01**	0.07

Values are based on multivariable linear regression models and reflect differences (95%CI) in individual cardiometabolic outcomes (age and sex adjusted SD scores) and in cardiometabolic score per 10 g/d increase in protein intake, and for tertiles of protein intake, as compared to the lowest tertile. Significant effect estimates are indicated in ***bold****.*

Protein intakes are energy-adjusted using the residual method. Tertiles are computed based on the total population for analysis (Figure 1, n=2,965).

Abbreviations: SDS, standard deviation score; BF%, body fat percentage; SBP, systolic blood pressure; DBP, diastolic blood pressure; HDL-C, high-density lipoprotein cholesterol.

Supplemental Table S5. Covariate-adjusted associations of animal and vegetable protein intake at the age of 1 year with cardiometabolic outcomes at 6 years.

	BF% (SDS)	Insulin (SDS)	SBP (SDS)	DBP (SDS)	HDL-C (SDS)	Triglycerides (SDS)	Cardiometabolic risk factor score
**Girls**	*n* = 1,487	*n* = 980	*n* = 1,426	*n* = 1,457	*n* = 1,457	*n* = 984	*n* = 982
**Animal protein intake (g)**							
Per 10 g/d	***0.07 (0.01, 0.13)***	***0.09 (0.00, 0.17)***	*-0.01 (-0.08, 0.05)*	*-0.02 (-0.08, 0.05)*	*-0.04 (-0.12, 0.04)*	*0.02 (-0.06, 0.11)*	*0.09 (-0.11, 0.30)*
Tertile 1	Reference	Reference	Reference	Reference	Reference	Reference	Reference
Tertile 2	0.02 (-0.08, 0.12)	-0.04 (-0.19, 0.11)	-0.08 (-0.20, 0.05)	-0.02 (-0.14, 0.10)	0.08 (-0.07, 0.23)	0.03 (-0.12, 0.18)	-0.21 (-0.60, 0.18)
Tertile 3	**0.10 (0.00, 0.21)**	**0.14 (0.00, 0.29)**	-0.01 (-0.14, 0.12)	-0.05 (-0.17, 0.08)	0.01 (-0.15, 0.16)	0.06 (-0.11, 0.22)	0.13 (-0.27, 0.54)
P for trend[#]	0.06	0.09	0.82	0.48	0.94	0.52	0.52
**Vegetable protein intake (g)**							
Per 10 g/d	*0.03 (-0.07, 0.13)*	***0.14 (0.00, 0.28)***	*-0.01 (-0.13, 0.11)*	*-0.04 (-0.15, 0.08)*	*0.12 (-0.03, 0.27)*	*-0.09 (-0.24, 0.06)*	*-0.20 (-0.58, 0.17)*
Tertile 1	Reference	Reference	Reference	Reference	Reference	Reference	Reference
Tertile 2	-0.02 (-0.13, 0.08)	0.14 (-0.01, 0.29)	-0.07 (-0.19, 0.05)	0.04 (-0.08, 0.16)	0.06 (-0.09, 0.21)	-0.08 (-0.24, 0.07)	-0.04 (-0.43, 0.35)
Tertile 3	-0.02 (-0.13, 0.09)	**0.16 (0.00, 0.32)**	-0.02 (-0.15, 0.11)	-0.05 (-0.18, 0.08)	0.08 (-0.08, 0.24)	-0.05 (-0.21, 0.11)	-0.12 (-0.53, 0.29)
P for trend[#]	0.72	**0.05**	0.73	0.50	0.35	0.53	0.58
**Boys**	*n* = 1,422	*n* = 1,016	*n* = 1,381	*n* = 1,384	*n* = 1,384	*n* = 1,017	*n* = 1,013
**Animal protein intake (g)**							
Per 10 g/d	*-0.01 (-0.06, 0.05)*	*-0.03 (-0.10, 0.05)*	*-0.02 (-0.08, 0.05)*	*-0.05 (-0.11, 0.01)*	*0.07 (-0.01, 0.14)*	***-0.11 (-0.18, -0.03)***	***-0.23 (-0.42, -0.04)***
Tertile 1	Reference	Reference	Reference	Reference	Reference	Reference	Reference
Tertile 2	-0.06 (-0.17, 0.05)	0.03 (-0.13, 0.18)	-0.02 (-0.14, 0.10)	-0.05 (-0.17, 0.08)	-0.01 (-0.17, 0.14)	-0.10 (-0.26, 0.05)	-0.23 (-0.62, 0.54)
Tertile 3	0.00 (-0.11, 0.11)	-0.04 (-0.19, 0.11)	0.00 (-0.12, 0.12)	-0.07 (-0.20, 0.06)	0.10 (-0.05, 0.26)	**-0.20 (-0.35, -0.05)**	-0.34 (-0.72, 0.04)
P for trend[#]	0.99	0.57	0.94	0.33	0.19	**<0.01**	0.08
**Vegetable protein intake (g)**							
Per 10 g/d	*-0.01 (-0.12, 0.10)*	*-0.05 (-0.21, 0.11)*	*0.02 (-0.10, 0.15)*	*-0.06 (-0.19, 0.07)*	*0.09 (-0.07, 0.25)*	*-0.07 (-0.22, 0.09)*	*-0.15 (-0.54, 0.24)*
Tertile 1	Reference	Reference	Reference	Reference	Reference	Reference	Reference
Tertile 2	-0.01 (-0.13, 0.10)	-0.05 (-0.21, 0.11)	0.10 (-0.02, 0.23)	**0.15 (0.01, 0.28)**	-0.05 (-0.21, 0.11)	-0.08 (-0.24, 0.08)	0.09 (-0.30, 0.49)
Tertile 3	-0.01 (-0.13, 0.10)	-0.03 (-0.19, 0.13)	0.08 (-0.05, 0.20)	-0.01 (-0.14, 0.12)	0.02 (-0.15, 0.18)	-0.05 (-0.21, 0.11)	-0.02 (-0.41, 0.37)
P for trend#	0.88	0.73	0.26	0.74	0.78	0.57	0.89

*Values are based on multivariable linear regression models and reflect differences (95%CI) in individual cardiometabolic outcomes (age and sex adjusted SD scores) and in cardiometabolic score score per 10 g/d increase in protein intake, and for tertiles of protein intake, as compared to the lowest tertile. Significant effect estimates are indicated in **bold**.*

Protein intakes are energy-adjusted using the residual method. Tertiles are computed based on the total population for analysis (Figure 1, n=2,965).

Models are adjusted for maternal age, BMI, education, and smoking during pregnancy; and child's ethnicity, birth weight Z-score, breastfeeding in the first four months of life, age at dietary measurement, energy intake, fat intake, height-for-age at 6y, participation in sports at 6y, and screen time at 6y. Models with animal protein intake are additionally adjusted for vegetable protein intake and vice versa.

[#] *Tests for trend were conducted using the tertiles of protein intake as a continuous variable.*

Abbreviations: SDS, standard deviation score; BF%, body fat percentage; SBP, systolic blood pressure; DBP, diastolic blood pressure; HDL-C, high-density lipoprotein cholesterol.

Supplemental Table S6. Covariate-adjusted associations of total protein intake at the age of 1 year with cardiometabolic outcomes at 6 years, in Dutch children only.

	BF% (SDS)	Insulin (SDS)	SBP (SDS)	DBP (SDS)	HDL-C (SDS)	Triglycerides (SDS)	Cardiometabolic risk factor score
**Whole group**	*n* = 1,957	*n* = 1,348	*n* = 1,925	*n* = 1,925	*n* = 1,355	*n* = 1,352	*n* = 1,289
Per 10 g/d	*0.04 (-0.01, 0.08)*	*0.02 (-0.05, 0.08)*	*-0.03 (-0.09, 0.02)*	*-0.06 (-0.11, 0.00)*	*0.02 (-0.05, 0.09)*	*-0.05 (-0.12, 0.02)*	-0.05 (-0.22, 0.12)
Tertile 1	Reference	Reference	Reference	Reference	Reference	Reference	Reference
Tertile 2	0.01 (-0.04, 0.19)	0.06 (-0.07, 0.19)	-0.10 (-0.20, 0.00)	**-0.11 (-0.21, -0.01)**	-0.05 (-0.18, 0.09)	0.01 (-0.13, 0.14)	-0.07 (-0.37, 0.24)
Tertile 3	0.06 (-0.03, 0.14)	0.01 (-0.12, 0.14)	-0.08 (-0.18, 0.03)	**-0.15 (-0.25, -0.05)**	0.04 (-0.09, 0.17)	-0.08 (-0.21, 0.06)	-0.25 (-0.56, 0.05)
P for trend[#]	0.12	0.85	0.14	**<0.01**	0.56	0.26	0.44
**Girls**	*n* = 984	*n* = 645	*n* = 971	*n* = 971	*n* = 649	*n* = 647	*n* = 613
Per 10 g/d	***0.06 (0.00, 0.13)***	***0.11 (0.01, 0.21)***	*-0.05 (-0.13, 0.03)*	*-0.06 (-0.13, 0.02)*	*0.02 (-0.08, 0.12)*	*-0.02 (-0.12, 0.08)*	0.00 (-0.25, 0.25)
Tertile 1	Reference	Reference	Reference	Reference	Reference	Reference	Reference
Tertile 2	0.08 (-0.04, 0.19)	0.09 (-0.10, 0.27)	-0.05 (-0.20, 0.09)	-0.07 (-0.21, 0.08)	-0.08 (-0.26, 0.11)	0.03 (-0.16, 0.22)	0.19 (-0.27 0.65)
Tertile 3	0.07 (-0.04, 0.19)	0.16 (-0.03, 0.35)	-0.10 (-0.25, 0.06)	-0.11 (-0.26, 0.03)	0.04 (-0.16, 0.23)	0.04 (-0.16, 0.23)	0.02 (-0.45, 0.50)
P for trend[#]	0.23	0.10	0.21	0.13	0.73	0.71	0.91
**Boys**	*n* = 973	*n* = 703	*n* = 954	*n* = 954	*n* = 706	*n* = 705	*n* = 676
Per 10 g/d	*0.00 (-0.06, 0.06)*	*-0.05 (-0.14, 0.04)*	*-0.02 (-0.09, 0.06)*	*-0.06 (-0.14, 0.01)*	*0.03 (-0.06, 0.12)*	***-0.09 (-0.18, 0.00)***	*-0.14 (-0.36, 0.08)*
Tertile 1	Reference	Reference	Reference	Reference	Reference	Reference	Reference
Tertile 2	-0.08 (-0.20, 0.04)	0.00 (-0.18, 0.18)	-0.14 (-0.28, 0.01)	**-0.15 (-0.30, 0.00)**	-0.02 (-0.20, 0.17)	-0.04 (-0.23, 0.14)	-0.21 (-0.65, 0.23)
Tertile 3	0.02 (-0.10, 0.14)	-0.11 (-0.29, 0.07)	-0.07 (-0.22, 0.08)	**-0.19 (-0.34, -0.04)**	0.05 (-0.14, 0.23)	**-0.20 (-0.38, -0.01)**	-0.33 (-0.76, 0.11)
P for trend[#]	0.73	0.21	0.36	**0.01**	0.61	**0.03**	0.14

*Values are based on multivariable linear regression models and reflect differences (95%CI) in individual cardiometabolic outcomes (age and sex adjusted SD scores) and in cardiometabolic score score per 10 g/d increase in protein intake, and for tertiles of protein intake, as compared to the lowest tertile. Significant effect estimates are indicated in **bold**.*

Protein intakes are energy-adjusted using the residual method. Tertiles are computed based on the total population for analysis (Figure 1, n=2,965).

Models are adjusted for maternal age, BMI, education, and smoking during pregnancy; and child's birth weight Z-score, breastfeeding in the first four months of life, age at dietary measurement, energy intake, fat intake, height-for-age at 6y, participation in sports at 6y, and screen time at 6y.

[#] Tests for trend were conducted using the tertiles of protein intake as a continuous variable.

Abbreviations: SDS, standard deviation score; BF%, body fat percentage; SBP, systolic blood pressure; DBP, diastolic blood pressure; HDL-C, high-density lipoprotein cholesterol.

Supplemental Table S7. Covariate-adjusted associations of protein intake at the age of 2 years with cardiometabolic outcomes at 6 years.

	BF% (SDS)	Insulin (SDS)	SBP (SDS)	DBP (SDS)	HDL-C (SDS)	Triglycerides (SDS)	Cardiometabolic risk factor score
**Whole group**	*n* = 698	*n* = 473	*n* = 685	*n* = 685	*n* = 475	*n* = 474	*n* = 450
Per 10 g/d	*-0.08 (-0.17, 0.02)*	*-0.03 (-0.19, 0.13)*	*-0.06 (-0.18, 0.07)*	*-0.09 (-0.22, 0.04)*	*0.03 (-0.14, 0.20)*	*-0.06 (-0.22, 0.11)*	-0.24 (-0.64, 0.16)
Tertile 1	Reference	Reference	Reference	Reference	Reference	Reference	Reference
Tertile 2	-0.07 (-0.21, 0.07)	0.05 (-0.17, 0.28)	-0.08 (-0.26, 0.11)	-0.08 (-0.26, 0.11)	0.01 (-0.24, 0.25)	-0.01 (-0.24, 0.23)	-0.29 (-0.87, 0.29)
Tertile 3	-0.14 (-0.29, 0.01)	-0.05 (-0.29, 0.18)	0.00 (-0.20, 0.19)	-0.11 (-0.31, 0.08)	0.04 (-0.21, 0.30)	-0.05 (-0.29, 0.20)	-0.24 (-0.84, 0.37)
P for trend[#]	0.06	0.67	0.96	0.25	0.73	0.70	0.44
**Girls**	*n* = 348	*n* = 236	*n* = 344	*n* = 344	*n* = 238	*n* = 238	*n* = 227
Per 10 g/d	*-0.05 (-0.19, 0.08)*	*0.00 (-0.22, 0.22)*	*-0.13 (-0.31, 0.05)*	*-0.11 (-0.28, 0.06)*	*-0.07 (-0.31, 0.17)*	*0.08 (-0.16, 0.31)*	-0.01 (-0.60, 0.57)
Tertile 1	Reference	Reference	Reference	Reference	Reference	Reference	Reference
Tertile 2	0.01 (-0.19, 0.20)	0.11 (-0.22, 0.43)	-0.24 (-0.01, 0.02)	-0.13 (-0.37, 0.12)	-0.29 (-0.64, 0.06)	0.08 (-0.27, 0.42)	-0.04 (-0.88, 0.80)
Tertile 3	-0.08 (-0.29, 0.13)	0.03 (-0.32, 0.38)	-0.10 (-0.38, 0.87)	-0.20 (-0.46, 0.06)	-0.07 (-0.43, 0.30)	0.16 (-0.20, 0.53)	0.00 (-0.91, 0.91)
P for trend[#]	0.45	0.19	0.45	0.13	0.69	0.38	0.99
**Boys**	*n* = 350	*n* = 237	*n* = 341	*n* = 341	*n* = 237	*n* = 236	*n* = 223
Per 10 g/d	*-0.12 (-0.26, 0.02)*	*-0.07 (-0.31, 0.17)*	*0.00 (-0.18, 0.17)*	*-0.09 (-0.28, 0.10)*	*0.19 (-0.07, 0.44)*	*-0.20 (-0.44, 0.04)*	***-0.60 (-1.20, 0.00)***
Tertile 1	Reference	Reference	Reference	Reference	Reference	Reference	Reference
Tertile 2	-0.16 (-0.37, 0.05)	0.04 (-0.29, 0.37)	0.06 (-0.20, 0.33)	-0.05 (-0.33, 0.24)	**0.38 (0.03, 0.73)**	-0.13 (-0.47, 0.20)	-0.62 (-1.47, 0.22)
Tertile 3	**-0.22 (-0.43, 0.00)**	-0.13 (-0.46, 0.21)	0.06 (-0.21, 0.33)	-0.05 (-0.34, 0.24)	0.18 (-0.18, 0.54)	-0.21 (-0.55, 0.14)	-0.50 (-1.36, 0.36)
P for trend[#]	**<0.05**	0.46	0.65	0.74	0.35	0.24	0.26

Values are based on multivariable linear regression models and reflect differences (95%CI) in individual cardiometabolic outcomes (age and sex adjusted SD scores) and in cardiometabolic score score per 10 g/d increase in protein intake, and for tertiles of protein intake, as compared to the lowest tertile. Significant effect estimates are indicated in ***bold****.*

Protein intakes are energy-adjusted using the residual method. Tertiles are computed based on the total population for analysis for diet at 2 years (Figure S1, n=714).

Models are adjusted for maternal age, BMI, education, and smoking during pregnancy; and child's ethnicity, birth weight Z-score, breastfeeding in the first four months of life, age at dietary measurement, energy intake at 2y, fat intake at 2y, height-for-age at 6y, participation in sports at 6y, and screen time at 6y.

[#] *Tests for trend were conducted using the tertiles of protein intake as a continuous variable.*

Abbreviations: SDS, standard deviation score; BF%, body fat percentage; SBP, systolic blood pressure; DBP, diastolic blood pressure; HDL-C, high-density lipoprotein cholesterol.
